# Supplementary material for: Nano-Sheet-like Morphology of Nitrogen-Doped Graphene-Oxide-Grafted Manganese Oxide and Polypyrrole Composite for Chemical Warfare Agent Simulant Detection
Source: Nanomaterials (Basel). 2022 Aug 27;12(17):2965. doi: 10.3390/nano12172965 (PMC9457797; doi:10.3390/nano12172965)
Supplement: Supplementary file 1 [file nanomaterials-12-02965-s001.zip › nanomaterials-1851425-supplementary.pdf]

nanomaterials

MDPI

Supplementary Materials

Nano-Sheet-Like Morphology of Nitrogen-Doped Graphene-Oxide-Grafted Manganese Oxide and Polypyrrole Composite for Chemical Warfare Agent Simulant Detection

Sanjeeb Lama [1], Bong-Gyu Bae [1], Sivalingam Ramesh [2], Young-Jun Lee [1], Namjin Kim [3] and Joo-Hyung Kim [1,*]

[1] Laboratory of Intelligent Devices and Thermal Control, Department of Mechanical Engineering, Inha University, Incheon 22212, Korea
[2] Department of Mechanical, Robotics and Energy Engineering, Dongguk University, Seoul 04620, Korea
[3] Department of Mechanical Engineering, Jeju National University, Jeju 63243, Korea
* Correspondence: joohyung.kim@inha.ac.kr

FIGURES:

Figure S1. Chemical warfare agents (a) Sarin, (b) Soman, (c) Tabun, and its simulant (d) DMMP. 5
Figure S2. The chemical structure of (a) NGO@MnO_2 (b) NGO@MnO_2/PPy. The figure is not to scale. 6
Figure S3. Schematic diagram of gas sensing system for QCM sensor. 8
Figure S4. Schematic diagram of gas sensing system for SAW sensor. 9
Figure S5. (a) QCM200 setup (digital controller, QCM crystals in Petri dish, crystal controller, and flow cell) (b) QCM flow cell (c) QCM signal monitoring program (d) Schematic of gas flow pattern in the flow cell. 10
Figure S6. (a) SAW sensor setup for the experimentation (b) Top view of the SAW sensor mounted on the test board (c) Front view of detection chamber with top cover for gas monitoring (d) Schematic of gas flow diagram in the detection chamber (e) SAW sensor monitoring program to check signals from SAW ports. 11
Figure S7. Schematic of (a) QCM crystal (SRS) (b) SAW sensor developed *in-house.* 12
Figure S8. (a), (b) FTIR analysis and (c), (d) XRD pattern, of NGO@MnO_2 (red), and NGO@MnO_2/PPy (Green). 13
Figure S9. XPS analysis of NGO@MnO_2. 14
Figure S10. XPS analysis of NGO@MnO_2/PPy. 15
Figure S11. FE-TEM images of NGO@MnO_2 with different magnifications. 16
Figure S12. FE-TEM images of NGO@MnO_2/PPy with different magnifications. 16
Figure S13. The effect of mass accumulation of (a) NGO@MnO_2 (b) NGO@MnO_2/PPy on the QCM sensor (1) adsorption (2) desorption (3) regeneration (T = 20 °C, R.H. = 25–30%). 17
Figure S14. Repeatability of (a) NGO@MnO_2, and (b) NGO@MnO_2/PPy in QCM sensor (T = 20 °C, R.H. = 25–30%). 18
Figure S15. Response and recovery times of (a) NGO@MnO_2 (b) NGO@MnO_2/PPy in detection of 75 ppm DMMP (T = 20 °C, R.H. = 25–30%). 19
Figure S16. Repeatability of (a) NGO@MnO_2, and (b) NGO@MnO_2/PPy in SAW sensor (T = 20 °C, R.H. = 25–30%). 22
Figure S17. Response and recovery times of (a) NGO@MnO_2, and (b) NGO@MnO_2/PPy in SAW sensor (T = 20 °C, R.H. = 25–30%). 23
Figure S18. The effect of different R.H. conditions on the SAW sensor coated with NGO@MnO_2/PPy under 25–30, 60–65, and 85–90% R.H. conditions at 20 °C for 75 and 150 ppm DMMP. 26
Figure S19. The stability test of NGO@MnO_2/PPy for the detection of 150 ppm DMMP for five consecutive days. 27

TABLES:

Table S1. List of acronyms. 4
Table S2. Coefficients used for various vapors in this study [1–5]. 7
Table S3. Summary of sensing materials and its performance in QCM sensor. 20
Table S4. Summary of sensing materials and its performance in SAW sensor. 24

Table S1. List of acronyms.

Acronyms	Definition	Acronyms	Definition
CWAs	Chemical warfare agents	IPA	Isopropyl alcohol
QCM	Quartz Crystal Microbalance	sccm	standard cubic centimeters per minute
SAW	Surface Acoustic Wave	MFCs	Mass Flow Controllers
DMMP	Dimethyl methyl phosphonate	ppm	parts per million
NGO	Nitrogen doped Graphene Oxide	Fc	Carrier gas flow rate
MnO_2	Manganese dioxide	Po	Outlet pressure
PPy	Polypyrrole	P_{th}	Thermodynamic pressure
VOCs	Volatile Organic Compounds	P_s	Saturated vapor pressure
MEMS	Micro-electromechanical system	F_d	dilution gas flow rate
CO_3O_4	Cobalt oxide	SRS	Stanford Research System
R.H.	Relative Humidity	VNA	Vector Network Analyzer
ZnO	Zinc Oxide	SMA	SubMiniature Version A Connector
M.W.	Molecular weight	Δf	Resonant frequency
FTIR	Fourier Transform Infrared Spectroscopy	C_f	Sensitivity factor
XRD	X-Ray Diffraction	Δm	mass change
XPS	X-ray Photoelectron Spectroscopy	IDTs	Interdigital Transducers
SEM	Scanning Electron Microscopy	F_c	Central frequency
TEM	Transmission Electron Microscopy	v	Velocity
H_2O_2	Hydrogen peroxide	λ	Wavelength
DD	Double distilled water	FE-SEM	Field Emission Scanning Electron Microscopy
$NaNO_3$	Sodium Nitrate	FE-TEM	Field Emission Transmission Electron Microscopy
H_2SO_4	Sulfuric acid	EDX	Energy Dispersive X-Ray Analysis
KOH	Potassium hydroxide	R^2	Coefficient of determination
$FeCl_3$	Ferric chloride	D	Coefficient of variation
$KMnO_4$	Potassium permanganate	δ	Standard deviation (δ)
NH_4OH	Ammonium hydroxide	k	Average
PTFE	PTFE = Polytetrafluorethylene	T_{90}	response time to attain 90 % of the equilibrium

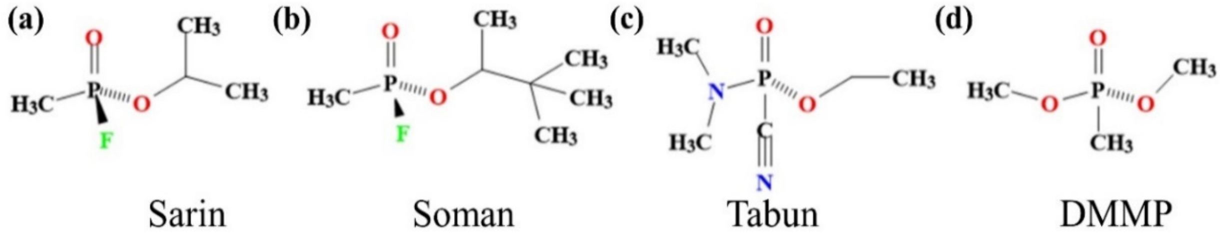

Figure S1. Chemical warfare agents (**a**) Sarin, (**b**) Soman, (**c**) Tabun, and its simulant (**d**) DMMP.

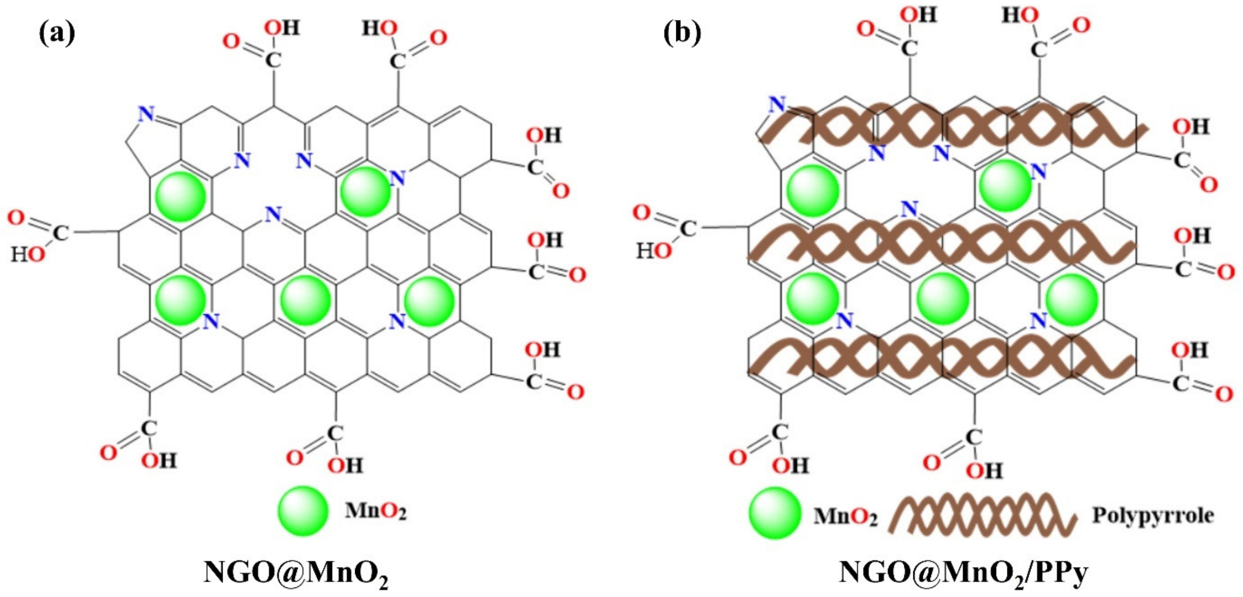

Figure S2. The chemical structure of (**a**) NGO@MnO_2 (**b**) NGO@MnO_2/PPy. The figure is not to scale.

Table S2. Coefficients used for various vapors in this study [1–5].

Vapors	Equation	Unit	*A*	*B*	*C*	Temp. Range [K]
DMMP	(2)	*T* (K), *P* (Pa)	22.31900	4340.00	−51.700	263.2–453.8
Ethanol	(3)	*T* (K), *P* (kPa)	7.24739	1599.04	−46.391	292.8–366.6
N-hexane	(3)	*T* (°C), *P* (mmHg)	6.87776	1171.53	224.366	286.2–342.7
Water	(3)	*T* (K), *P* (bar)	5.40221	1838.67	−31.737	273.0–303.0
Toluene	(3)	*T* (°C), *P* (mmHg)	6.95464	1344.80	219.482	279.0–409.0
Methanol	(3)	*T* (°C), *P* (mmHg)	8.07240	1574.99	238.870	257.0–364.0

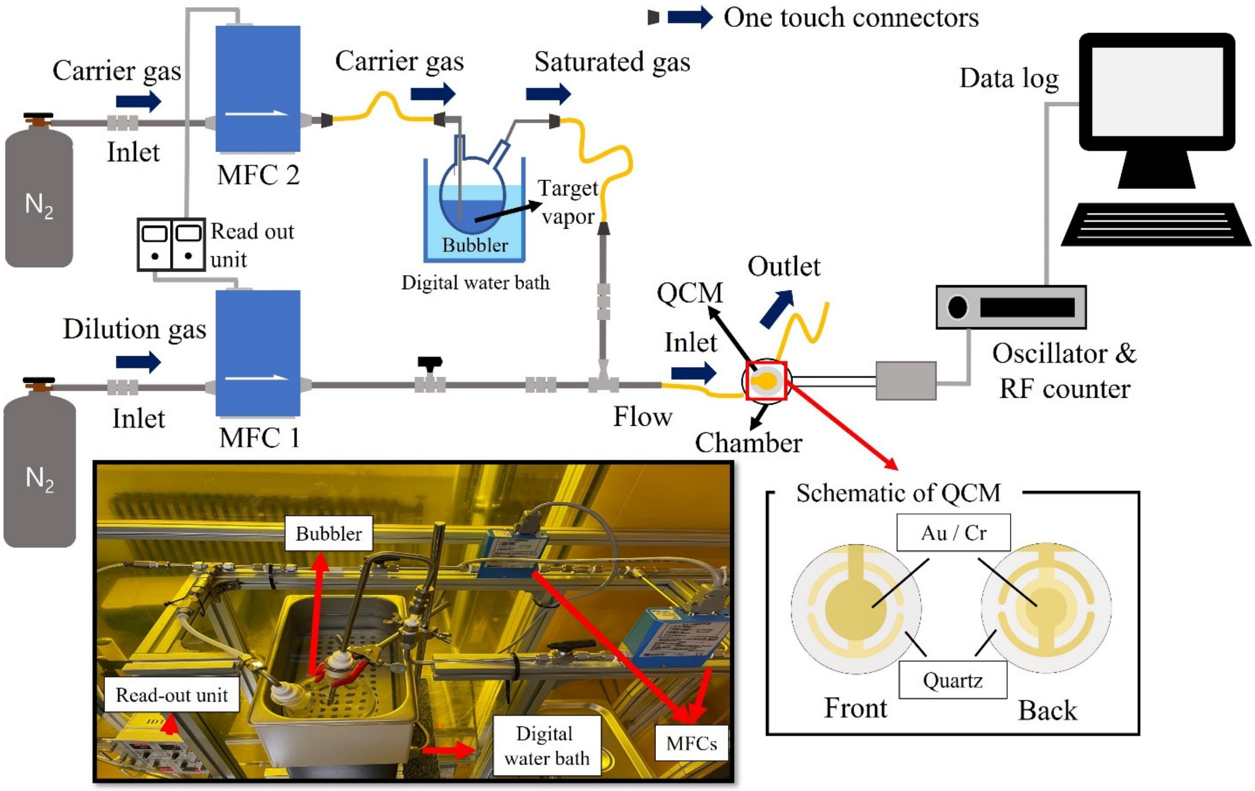

Figure S3. Schematic diagram of gas sensing system for QCM sensor.

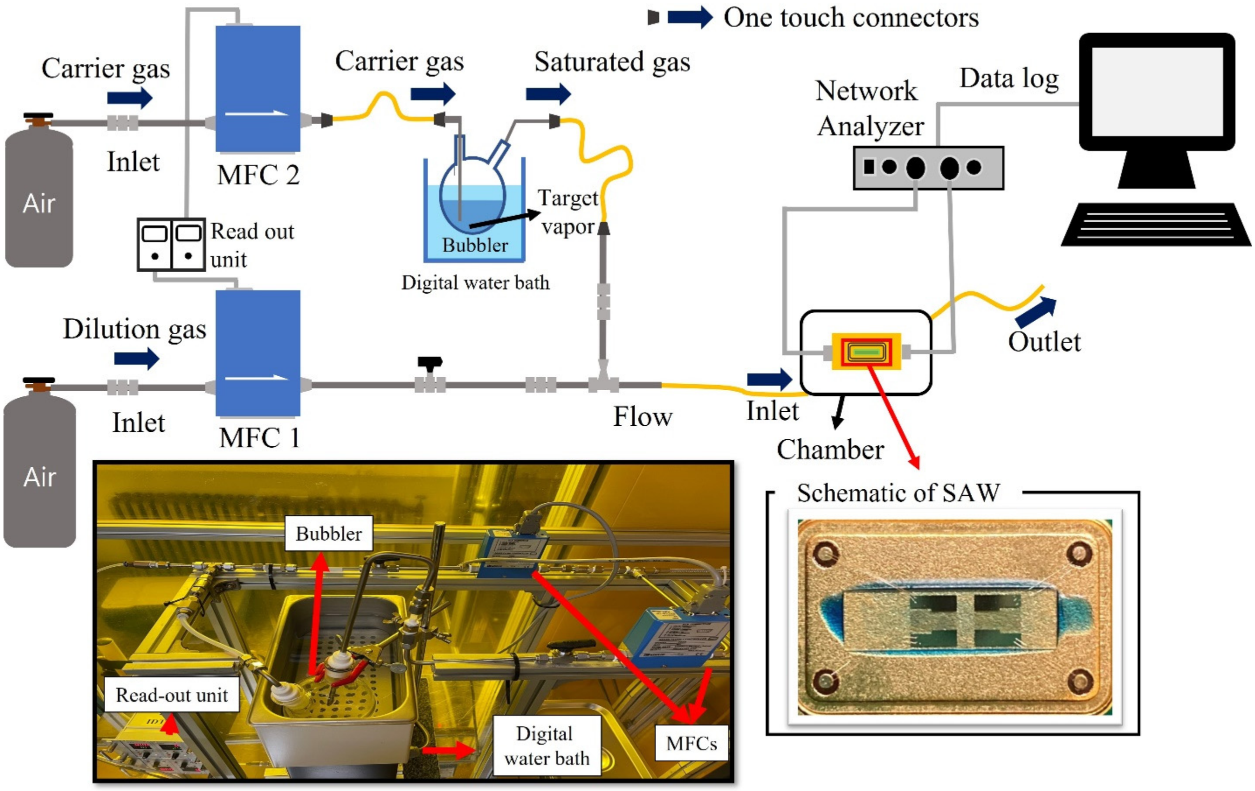

Figure S4. Schematic diagram of gas sensing system for SAW sensor.

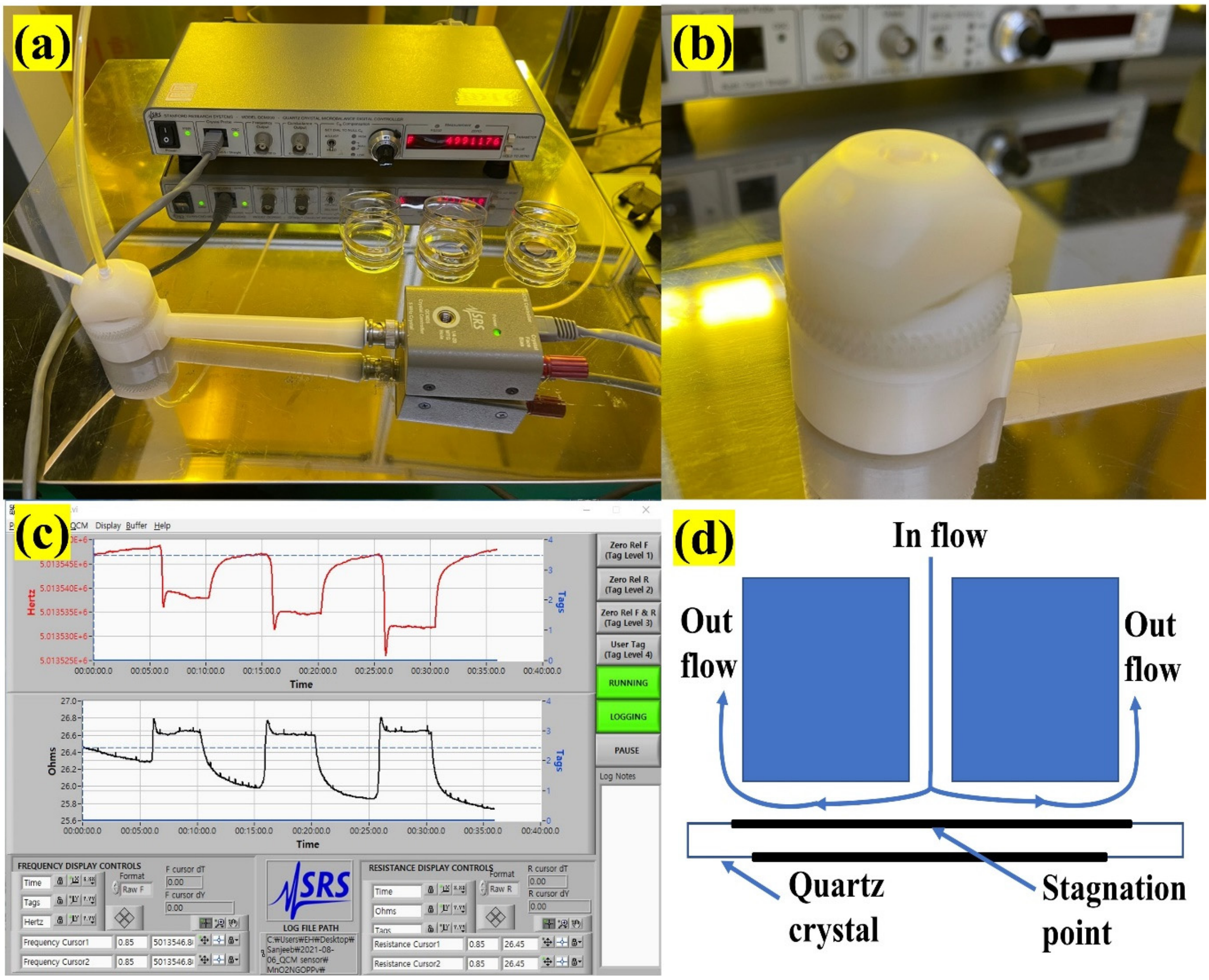

Figure S5. (**a**) QCM200 setup (digital controller, QCM crystals in Petri dish, crystal controller, and flow cell) (**b**) QCM flow cell (**c**) QCM signal monitoring program (**d**) Schematic of gas flow pattern in the flow cell.

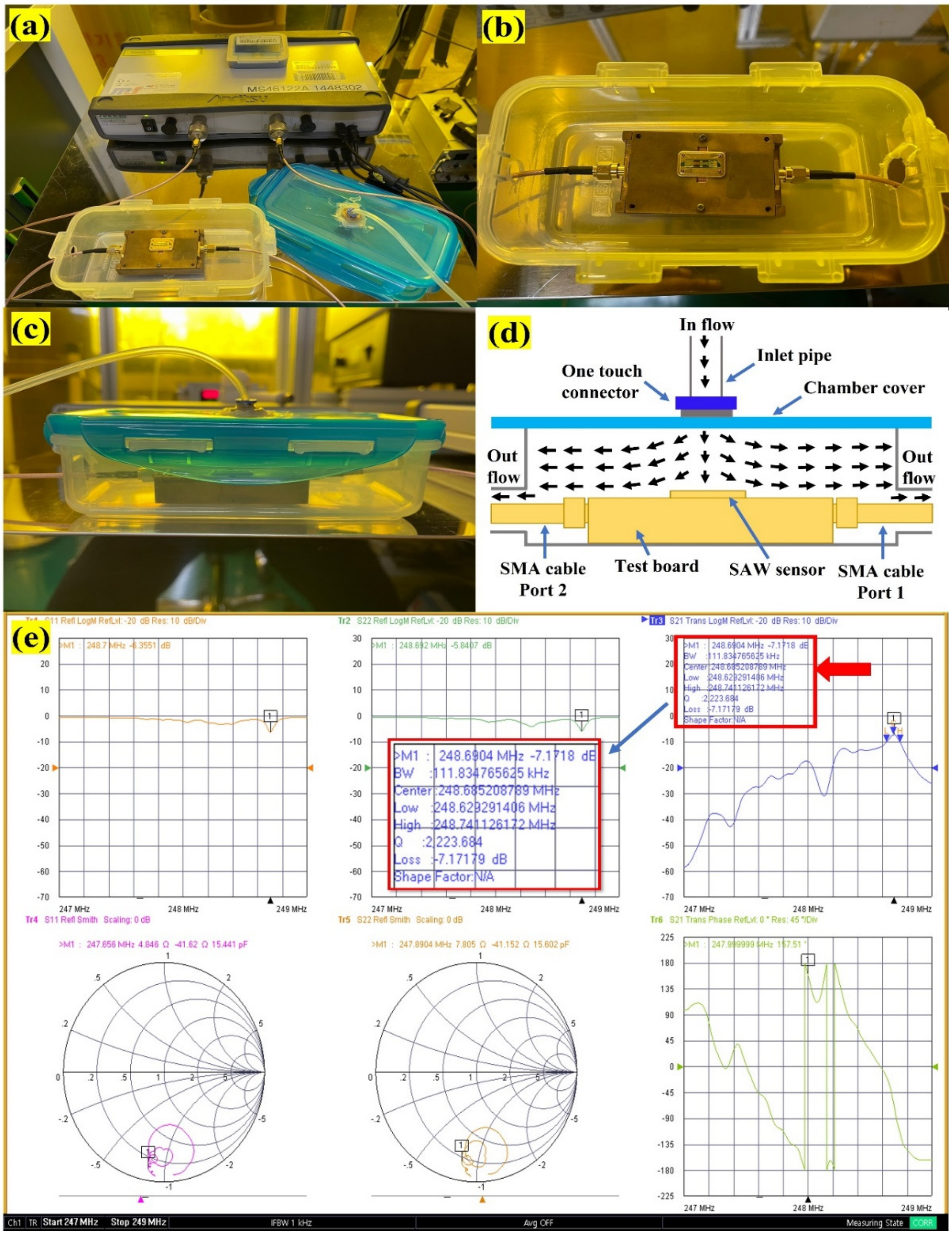

Figure S6. (**a**) SAW sensor setup for the experimentation (**b**) Top view of the SAW sensor mounted on the test board (**c**) Front view of detection chamber with top cover for gas monitoring (**d**) Schematic of gas flow diagram in the detection chamber (**e**) SAW sensor monitoring program to check signals from SAW ports.

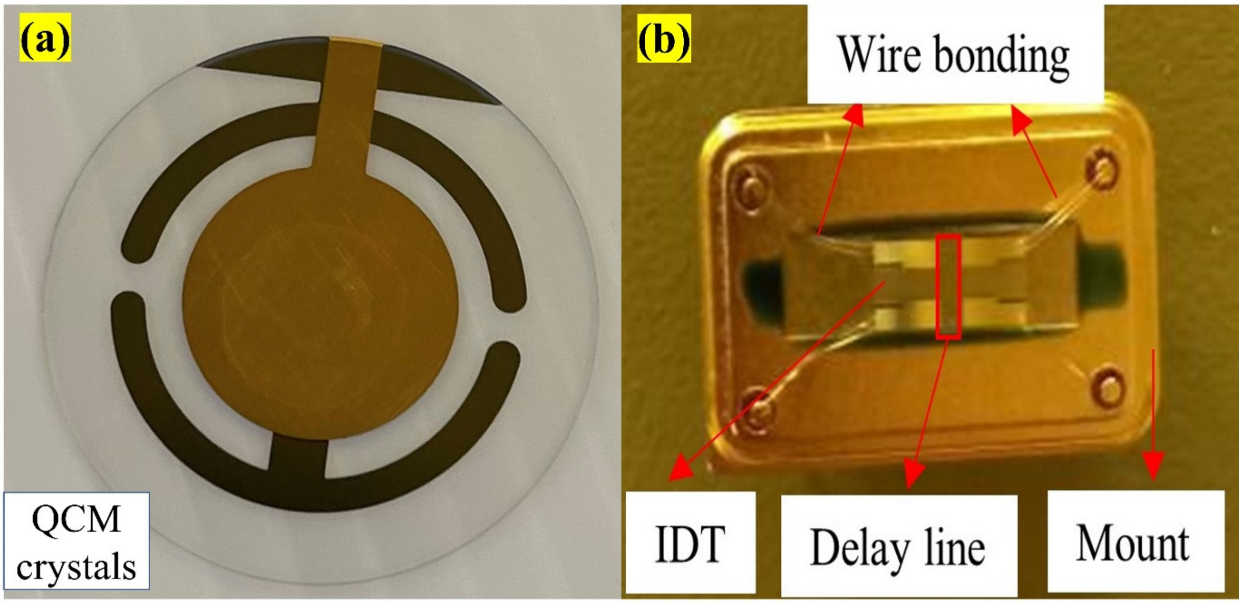

Figure S7. Schematic of (**a**) QCM crystal (SRS) (**b**) SAW sensor developed *in-house*.

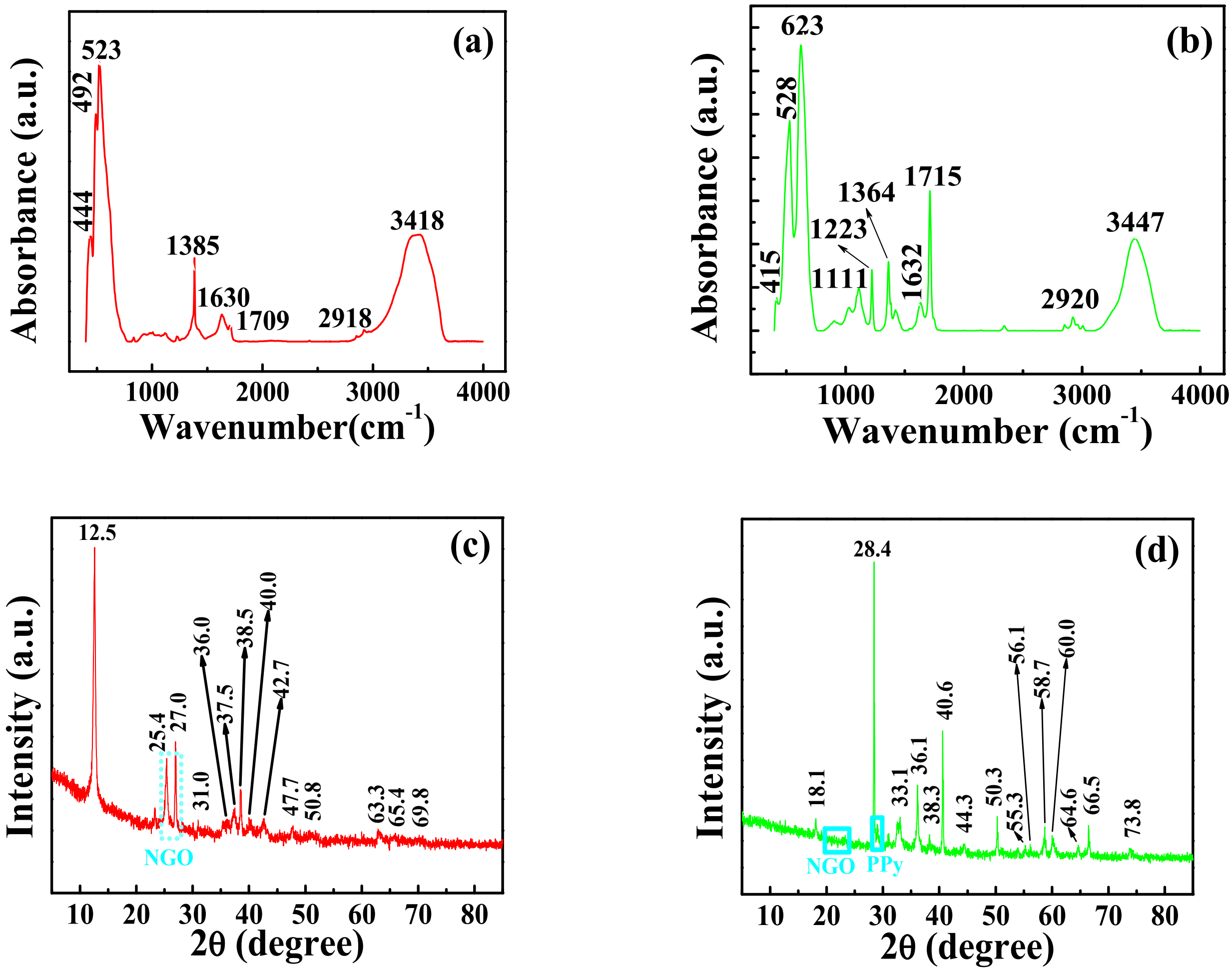

Figure S8. (**a**,**b**) FTIR analysis and (**c**,**d**) XRD pattern, of NGO@MnO_2 (red), and NGO@MnO_2/PPy (Green).

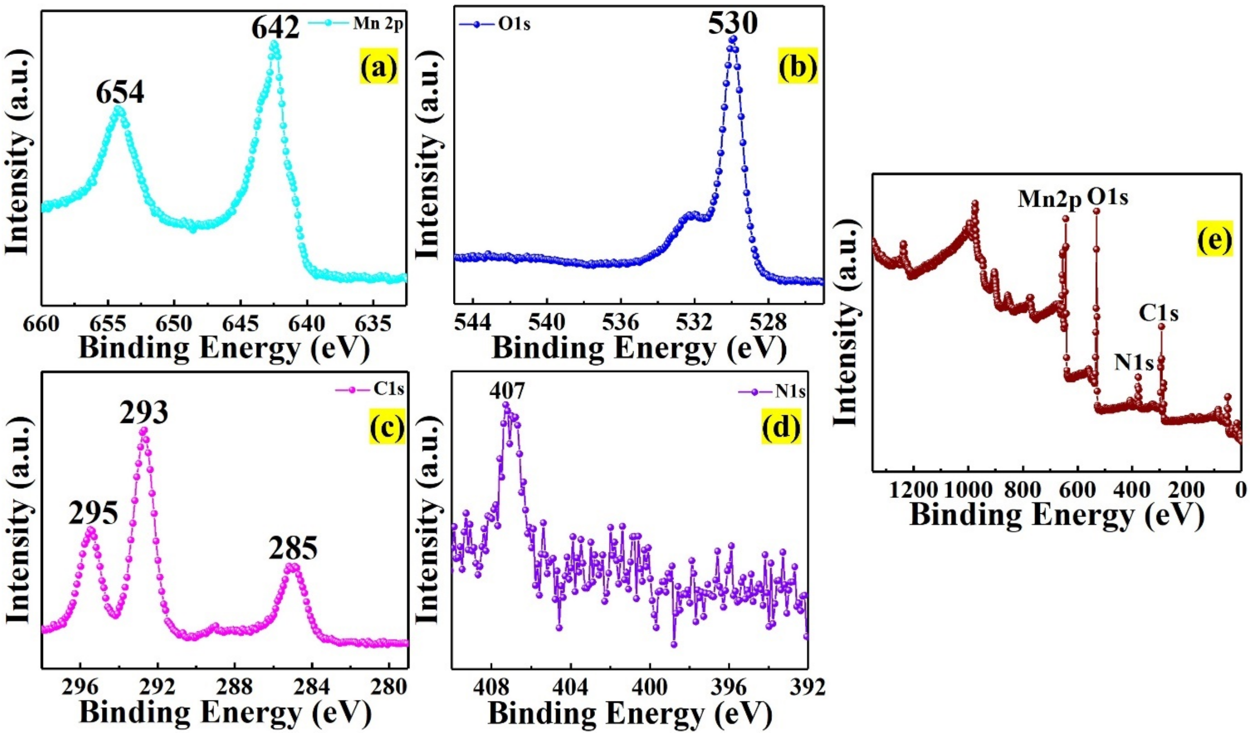

Figure S9. XPS analysis of NGO@MnO_2 composite. (**a**) Mn 2p, (**b**) O 1s, (**c**) C 1s and (**d**) N 1s binding energy and (**e**) survey spectrum.

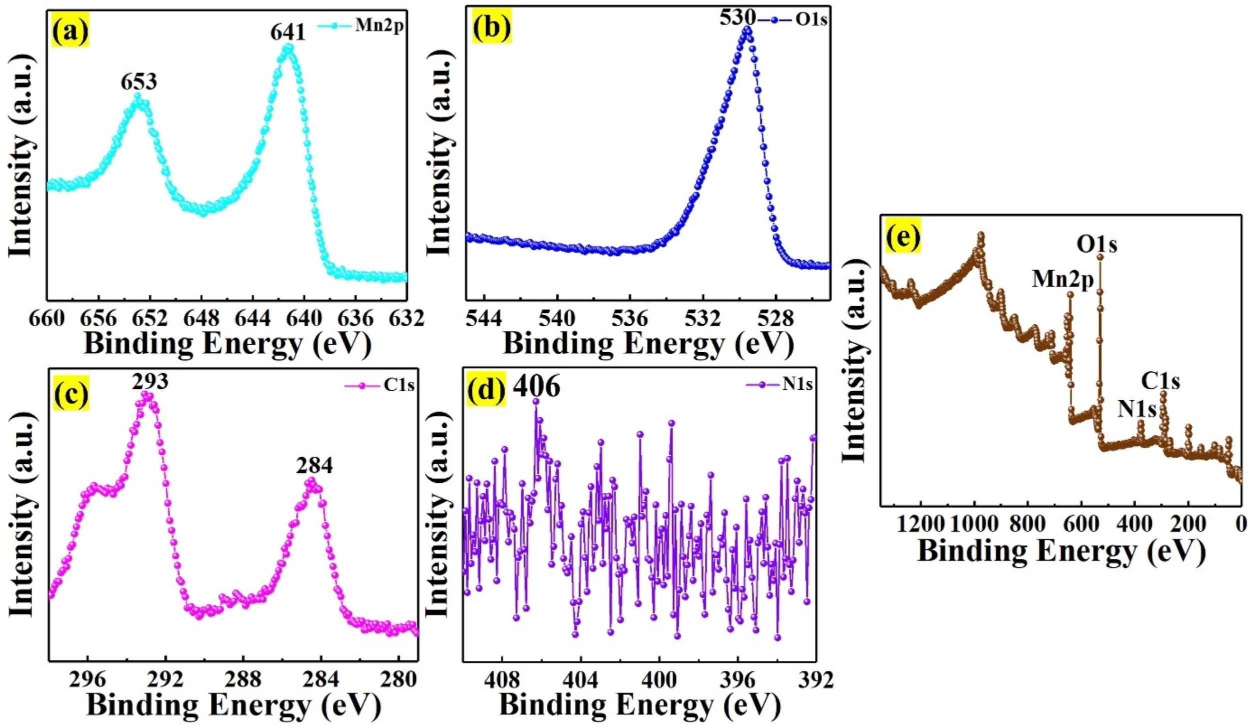

Figure S10. XPS analysis of NGO@MnO_2/PPy composites. (**a**) Mn 2p, (**b**) O 1s, (**c**) C 1s and (**d**) N 1s binding energy and (**e**) survey spectrum.

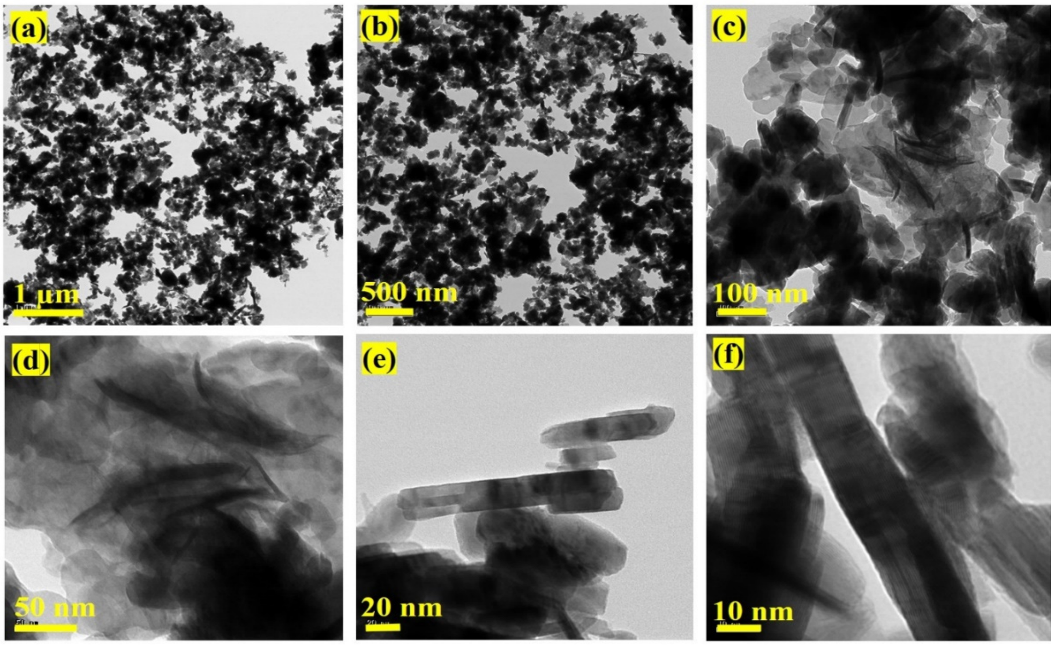

Figure S11. FE-TEM images of NGO@MnO_2 at the magnification of (**a**) 1 μm, (**b**) 500 nm, (**c**) 100 nm (**d**) 50 nm, (**e**) 20 nm, and (**f**) 10 nm.

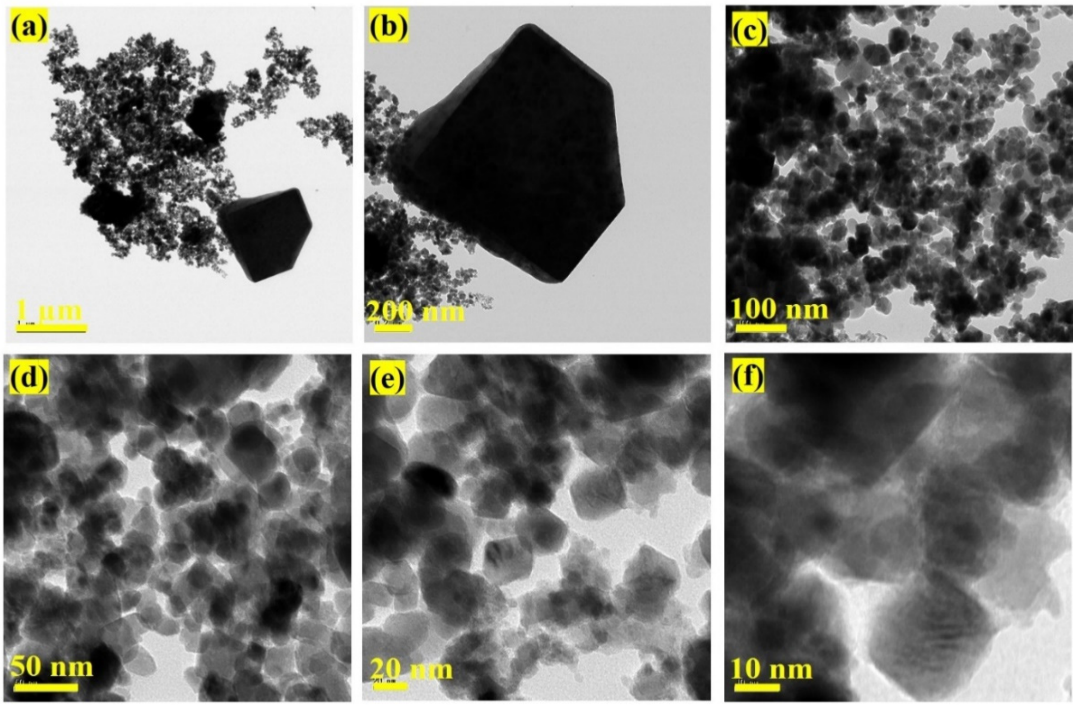

Figure S12. FE-TEM images of NGO@MnO_2/PPy at the magnification of (**a**) 1 μm, (**b**) 200 nm, (**c**) 100 nm (**d**) 50 nm, (**e**) 20 nm, and (**f**) 10 nm.

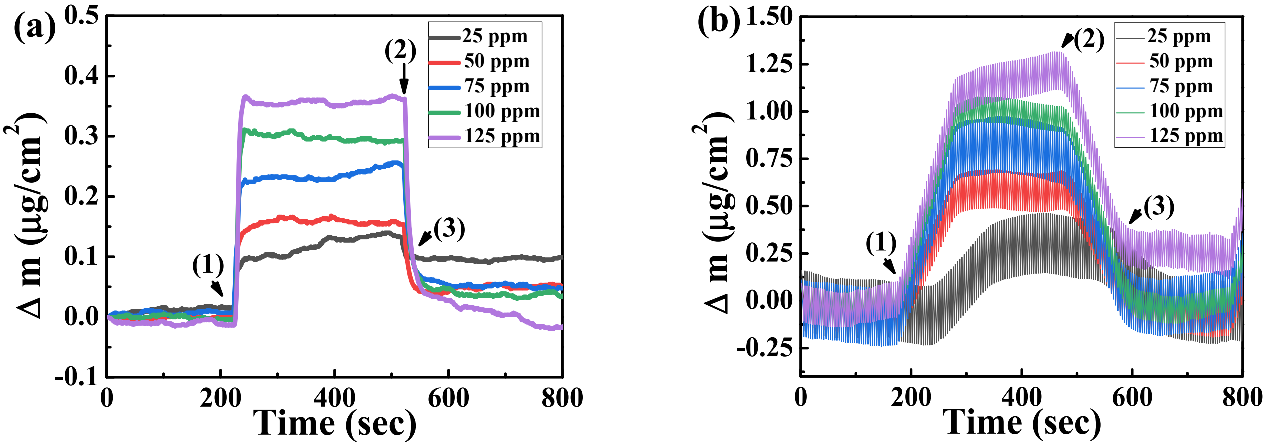

Figure S13. The effect of mass accumulation of (**a**) NGO@MnO_2 (**b**) NGO@MnO_2/PPy on the QCM sensor (1) adsorption (2) desorption (3) regeneration (T = 20 °C, R.H. = 25–30%).

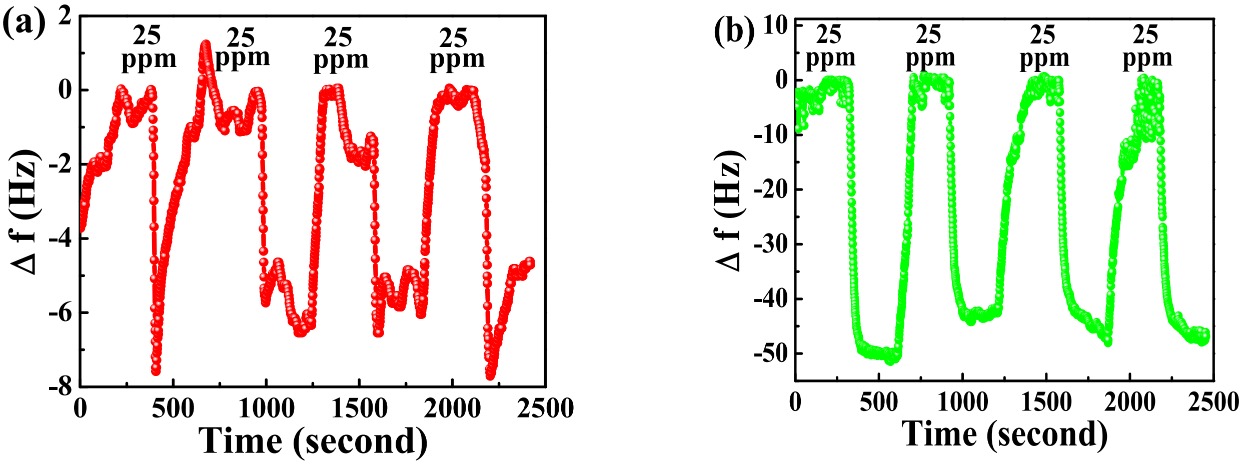

Figure S14. Repeatability of (**a**) NGO@MnO_2, and (**b**) NGO@MnO_2/PPy in QCM sensor (T = 20 °C, R.H. = 25–30%).

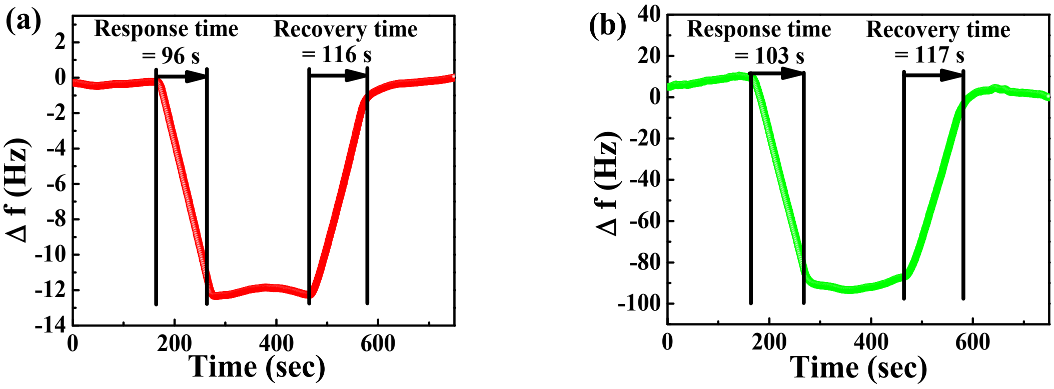

(**a**) (**b**)

Figure S15. Response and recovery times of (**a**) NGO@MnO_2 (**b**) NGO@MnO_2/PPy in detection of 75 ppm DMMP (T = 20 °C, R.H. = 25–30%).

Table S3. Summary of sensing materials and its performance in QCM sensor.

Ref.	Sensing Materials	Concentration	Response (Hz)	Response Time (s)	Recovery Time (s)
[6]	Acetylcholinesterase	20 mg/m^3	~4.5	-	-
[7]	HFIPP-GR	5 ppm	71 ± 4	T_{80} < 108	T_{80} = 600
[8]	PMDFPS	40 ppm	~600	-	-
[9]	WO_3 nanoflakes	3.91 ppm	<160	30	73
[10]	WO_3 particles	4 ppm	281	153	-
[11]	Cu-ZSM-5 (5)	40 ppm	~410	T_{80} < 100	T_{90} > 1800
[12]	ZnPcs-HF$_4$O-Cl (1b)	12 ppm	~175	T_{90} = 1080	-
[13]	TiO_2	10 ppm	~45	27	31
[14]	MIP	100 ppm	~80	-	-
[15]	TiO_2-SiO_2-HFIP (S4)	60 ppm	~575	< 60	< 60
[16]	U3/SBA-15	1 ppm	398	44	66
[17]	ZSM-5 zeolite	20 ppm	120	< 100	-
[18]	Ag^+ZSM-5	5 ppm	35	< 80	-
[19]	Hexafluoroisopropanol functionalized siloxane	50 ppm	~1150	T_{80} = 90	~175
[20]	ZrO_2 particles	1.5 ppb	~32.5	20	40
[21]	TU-4	60 ppm	3910	-	-
[22]	HFIP/SBA-15	60 ppm	~1600	-	-
[23]	Co_3O_4@gold /MWCNT/ PPy	60 ppm	90	T_{98} = 60	T_{98} = 493
[24]	TU13	60 ppm	3200	-	-
[25]	PMTFMPS	50 ppm	~1400	T_{80} = 60–80	170
[26]	Nano-SnO_2	5 ppm	450	~10	-
[27]	BHPHFP functionalized MCM-41	140 ppb	~60	-	-
[28]	V_2O_5 coated ZnO nanorods (S4)	15 ppm	~40	T_{80} < 300	T_{80} > 900
[29]	PVDF	300 ppm	~145	~60	~60
[30]	POSS	120 ppm	~2500	-	-
[31]	Fluorosiloxane	150 mg/m^3	~220	-	-
[32]	*Vic*-Dioximes (O4C)	12 ppm	~3000	T_{90} ~ 780	T_{90} ~ 1200
[33]	ZnO-modified NW- structured MnO2	1.75 ppm	~200	300	400
[34]	Hollow Ball-Like Nano-Fe_2O_3	4 ppm	115	25	-
[35]	NSrGO-HFHPB (1:4)	60 ppm	83	T_{98} = 22	T_{98} = 27
[36]	HFHPB-GQD	4 ppm	~10	T_{90} = 31	T_{90} = 34
[37]	PVDF	40 ppm	~140	-	-
This work	NGO@MnO_2	75 ppm	14	T_{90} = 96	T_{90} = 116
	NGO@MnO_2/PPy		107	T_{90} = 103	T_{90} = 117

Table S3 summarizes the various sensing materials and their performance in a QCM sensor. From this table, we observe that the NGO@MnO_2/PPy shows similar or greater frequency response compared to the materials given in Refs. [14,23,29,35,37], similar or shorter response time compared to the materials given in Refs. [7,10–12,19,25,28,32,33], and similar or shorter recovery time compared to the materials given in Refs. [7,11,19,23,25,28,32,33]. This excellent performance of NGO@MnO_2/PPy may arise from the increased surface area, higher conductivity from π-electron conjugation, the formation of hydrogen bond or Van der Waals forces, and good cohesive contact between the materials and electrodes [38–46]. The present work is not comparable to the reported works in the literature [7,9,10,12,13,16,18,20,26,27,32–34,36] in terms of frequency responses since

they all tested the materials in relatively lower concentrations. It is also important to note here that the present work is focused in DMMP detection ranging from 25 to 150 ppm. To add more to the comparisons, we notice the following. For the case of response time, some works [9,13,20,26,34,35] in the literature reported very fast response times (≤30 s) while some others showed the long response at low ppm and the short response at high ppm [8]. Response times of 5 min ≤ $T_{response}$ < 10 min [24,28,31,33,37], and ≥10 min [12,14,27,32] were also reported in the literature. Similar observation is also made for the recovery times. Some of the sensing materials exhibited fast recovery (≤ 40 s) [13,20,35,36], whereas some other materials exhibited long recovery times of 5 min ≤ $T_{recovery}$ < 10 min [23,24,31,33,37] and ≥10 min [7,11,12,14,27,28,32]. The literature also reports an incomplete recovery [10], and a case in which it was difficult to desorb [17].

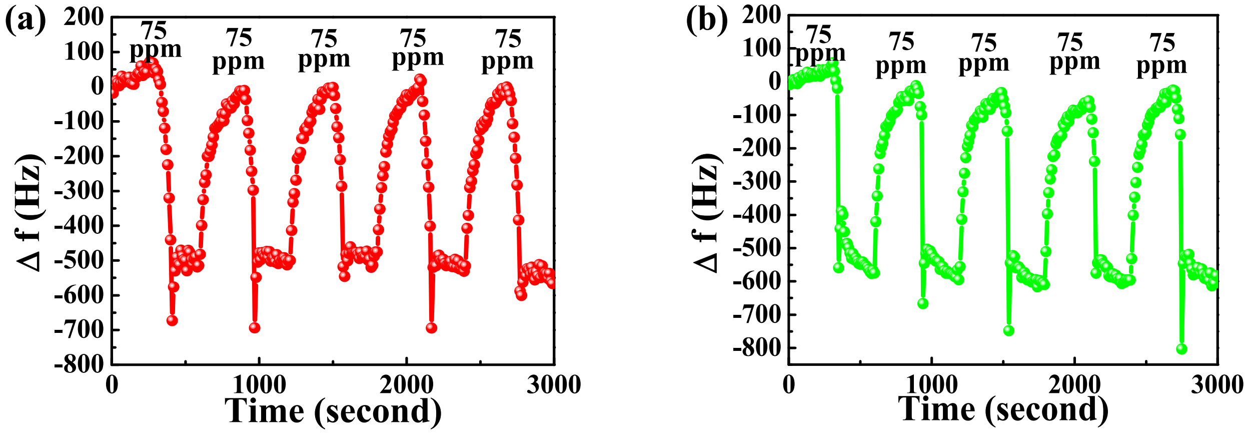

Figure S16. Repeatability of (**a**) NGO@MnO$_2$, and (**b**) NGO@MnO$_2$/PPy in SAW sensor (T = 20 °C, R.H. = 25–30%).

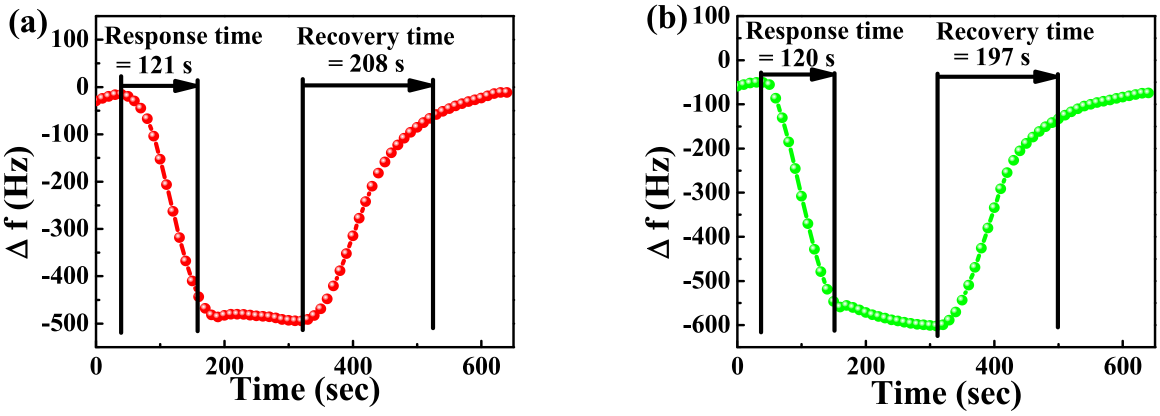

(**a**) (**b**)

Figure S17. Response and recovery times of (**a**) NGO@MnO$_2$, and (**b**) NGO@MnO$_2$/PPy in SAW sensor (T = 20 °C, R.H. = 25–30%).

Table S4. Summary of sensing materials and its performance in SAW sensor.

Ref.	Sensing Materials	Center Frequency (MHz)	Concentration	Response (Hz)	Response Time (s)	Recovery Time (s)
[47]	PMPS	434	20 ppm	~150,000	T_{80} ~ 30	-
[48]	Diamond nanoparticles	433	0.5 ppm	2400	~60	-
[49]	PIB	264	5 ppm	5369	-	-
[50]	MIP	300	50 mg/m^3	14000	1200	1200
[51]	PCPMS	157	1 ppm	~ 2300	300-600	1800
[52]	Carbowax	163	1 ppm	~ 14,000	T_{50} ~ 60	T_{50} ~ 240
[53]	SnO_2	433.9	50 ppm	4210	-	-
[54]	ZnO	433.9	100 ppb	355	-	-
[55]	ZnO (80 nm)	433.9	1600 ppm	52,000	37	720
[56]	SXFA (24 °C)	150	5.0 mg/m^3	5833	20	37
[57]	PMFOS	194	10 ppm	6400	-	-
[58]	SXFA	433.9	10 ppm	8070	-	-
[59]	Bisphenol	70	0.5 ppm	700	15	-
[60]	SXFA	150	200 mg/m^3	~38,000	T_{75} = 300	T_{75} < 1200
[61]	ZnO QDs	150	91 ppm	~3000	~120	~120
[62]	DKAP	434	20 mg/m^3	~80,000	T_{80} ~ 40	T_{80} ~ 25
[63]	Calixarene (20 °C)	300	20 mg/m^3	2150	174	80
[64]	Polymer 4	500	0.5 mg/m^3	3629	300	-
[65]	25-(thioalkyl-alkoxy)-p-tertbutylcalix [4] arene	300	5 mg/m^3	6041	229	-
[66]	HB-PE-COOH 2	500	0.05 ppm	5292	300	300
[67]	1-benzyl-3-phenylthiourea	250	98.6 ppm	51,200	260	470
[68]	Graphene/PVDF	433	10 ppm	~ 30,000	T_{90} ~ 4.5	T_{90} ~ 5.8
[69]	SXFA	158	1000 mg/m^3	~ 35,000	10	<10
[70]	PLF	434	1 mg/m^3	3100	T_{80} < 50	180
[71]	PEI with AchE	~ 69	6 ppm	65,000	90-100	-
[72]	POSS	250	507 mg/m^3	12,600	T_{80} = 22	-
[73]	LSFA	434	1 mg/m^3	10,000	T_{80} < 20	-
[74]	LaBHA	78	5 ppm	140	T_{80} = 240	T_{80} = 780
This work	NGO@MnO_2	250	150 ppm	1129	T_{90} = 121	T_{90} = 208
	NGO@MnO_2/PPy			1546	T_{90} = 120	T_{90} = 197

Table S4 summarizes the various sensing materials and their performance in a SAW sensor. The composite materials that we have developed are compared to other sensing materials in SAW sensors to find that the fabricated composite materials excelled with similar or shorter response time than the materials in the Refs. [50–53,57,60,63–67,74] and similar or shorter recovery time than the materials in the Refs. [47,50,51–55,57,60,65–67,72,74]. This exceptional response of the fabricated materials may have resulted from the sensitive behavior of MnO_2, NGO, and PPy, increment of free and mobile hole charge carriers, increased surface area, and the formation of hydrogen bond [39,42,46,75–77]. It is also interesting to note that some reported works [47,56,59,68,69,72,73] in literature showed short response time (≤ 30 s). The literature also reports response times of 5 min ≤ $T_{response}$ < 10 min [51,53,57,60,64,66] and ≥ 10 min [50]. While studying recovery time, some works [56,62,68,69] report short recovery time (≤ 40 s). Similarly, there are reports for longer recovery time of 5 min ≤ $T_{recovery}$ < 10 min [57,66,67] and ≥ 10 min [50,51,53,55,60,74]

as well. There are cases [58,65] in which the recovery time was longer than response time. Some studies [48,49,64,71] have not reported information on recovery time which is an important observation from the literature.

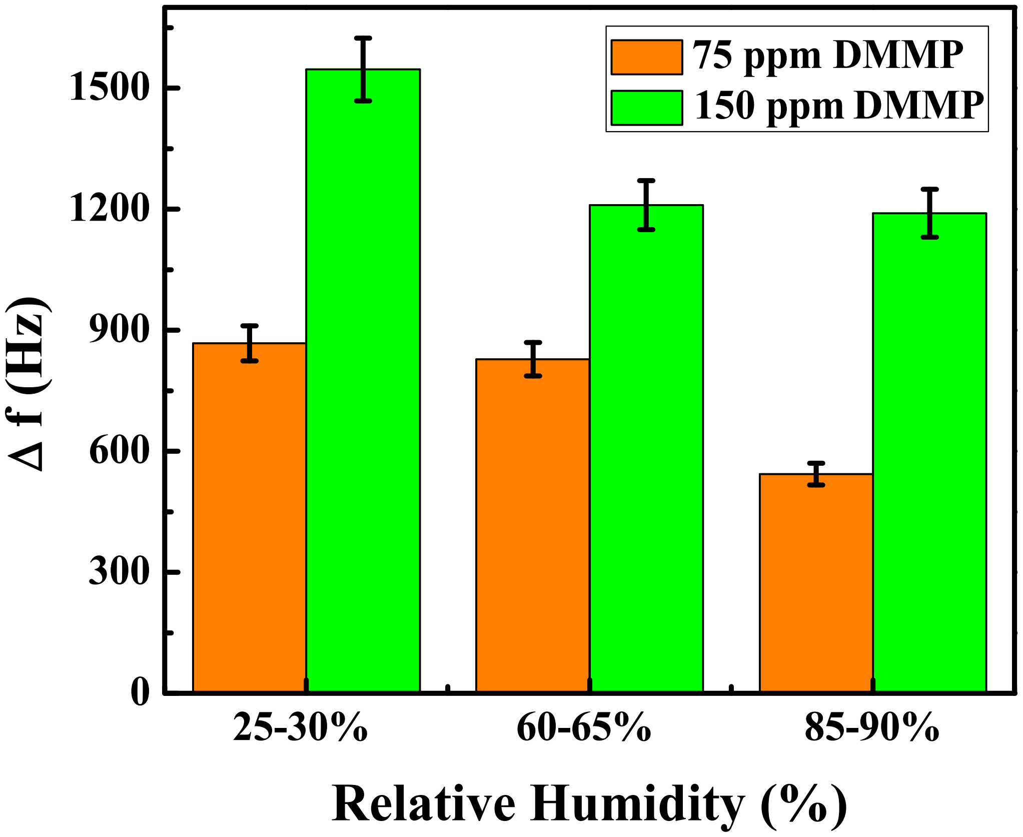

Figure S18. The effect of different R.H. conditions on the SAW sensor coated with NGO@MnO_2/PPy under 25–30, 60–65, and 85–90% R.H. conditions at 20 °C for 75 and 150 ppm DMMP.

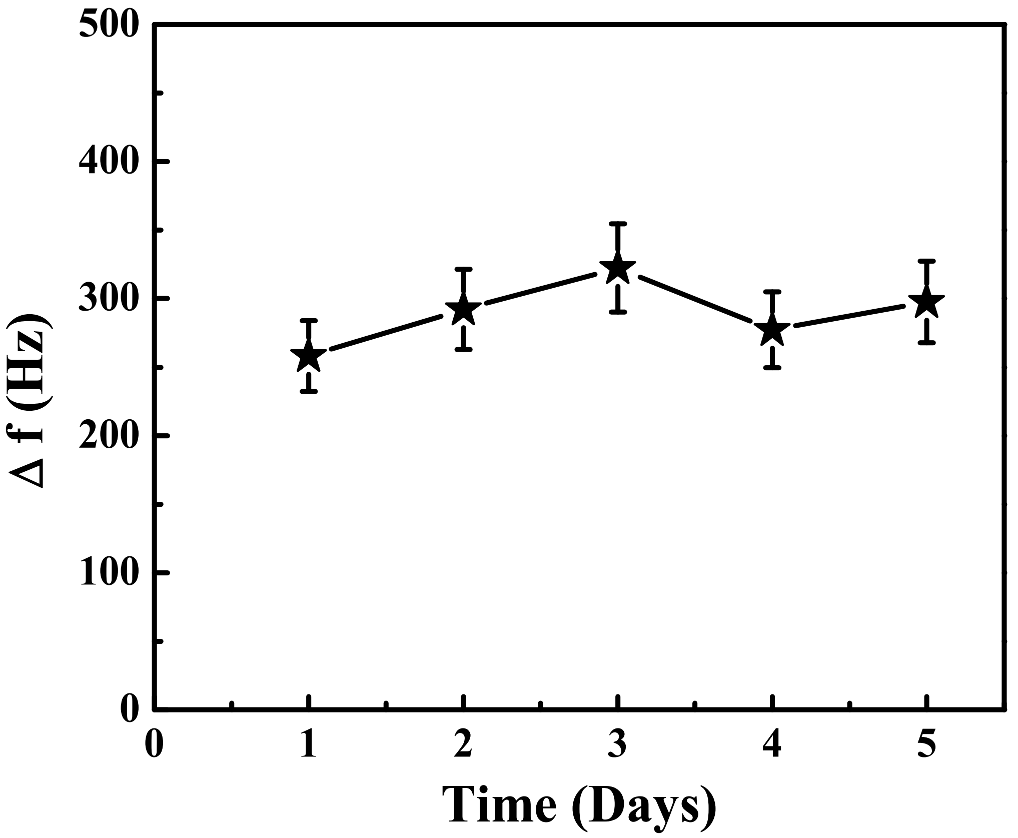

Figure S19. The stability test of NGO@MnO_2/PPy for the detection of 150 ppm DMMP for five consecutive days.

References

1. Butrow, A.B.; Buchanan, J.H.; Tevault, D.E. Vapor pressure of organophosphorus nerve agent simulant compounds. *J. Chem. Eng. Data* **2009**, *54*, 1876–1883.
2. Ambrose, D.; Sprake, C.H.S. Thermodynamic properties of organic oxygen compounds XXV. Vapour pressures and normal boiling temperatures of aliphatic alcohols. *J. Chem. Thermodyn.* **1970**, *2*, 631–645.
3. Willingham, C.B.; Taylor, W.J.; Pignocco, J.M.; Rossini, F.D. Vapor pressures and boiling points of some paraffin, alkylcyclopentane, alkylcyclohexane, and alkylbenzene hydrocarbons. *J. Res. Natl. Bur. Stand. (1934).* **1945**, *35*, 219–244.
4. Yaws, C.L.; Yang, H.C. To estimate vapor pressure easily. *Hydrocarb. Process.* **1989**, *68*.
5. Bridgeman, O.C.; Aldrich, E.W. Vapor pressure tables for water. **1964**.
6. Ma, W.; Tang, S.; Wei, Y.; Xie, G. Simple biosensing method to detect DMMP based on QCM transducer and acetylcholine esterase sensitive film. *Micro Nano Lett.* **2017**, *12*, 113–116.
7. Shaik, M.; Rao, V.K.; Ramana, G. V; Halder, M.; Gutch, P.K.; Pandey, P.; Jain, R. p-Hexafluoroisopropanol phenyl functionalized graphene for QCM based detection of dimethyl methylphosphonate, a simulant of the nerve agent sarin. *RSC Adv.* **2018**, *8*, 8240–8245.
8. He, W.; Liu, Z.; Du, X.; Jiang, Y.; Xiao, D. Analytical application of poly {methyl [3-(2-hydroxy-3, 4-difluoro) phenyl] propyl siloxane} as a QCM coating for DMMP detection. *Talanta* **2008**, *76*, 698–702.
9. Zhao, Y.; He, J.; Yang, M.; Gao, S.; Zuo, G.; Yan, C.; Cheng, Z. Single crystal WO_3 nanoflakes as quartz crystal microbalance sensing layer for ultrafast detection of trace sarin simulant. *Anal. Chim. Acta* **2009**, *654*, 120–126.
10. Zhao, Y.; Chen, H.; Wang, X.; He, J.; Yu, Y.; He, H. Flower-like tungsten oxide particles: Synthesis, characterization and dimethyl methylphosphonate sensing properties. *Anal. Chim. Acta* **2010**, *675*, 36–41.
11. Ji, X.; Yao, W.; Peng, J.; Ren, N.; Zhou, J.; Huang, Y. Evaluation of Cu-ZSM-5 zeolites as QCM sensor coatings for DMMP detection. *Sens. Actuators B: Chem.* **2012**, *166*, 50–55.
12. Tasaltin, C.; Gurol, I.; Harbeck, M.; Musluoglu, E.; Ahsen, V.; Ozturk, Z.Z. Synthesis and DMMP sensing properties of fluoroalkyloxy and fluoroaryloxy substituted phthalocyanines in acoustic sensors. *Sens. Actuators B: Chem.* **2010**, *150*, 781–787.

13. Zhao, Y.; Du, X.; Wang, X.; He, J.; Yu, Y.; He, H. Effects of F doping on TiO_2 acidic sites and their application in QCM based gas sensors. *Sens. Actuators B: Chem.* **2010**, *151*, 205–211.
14. Findeisen, A.; Wackerlig, J.; Samardzic, R.; Pitkänen, J.; Anttalainen, O.; Dickert, F.L.; Lieberzeit, P.A. Artificial receptor layers for detecting chemical and biological agent mimics. *Sens. Actuators B: Chem.* **2012**, *170*, 196–200.
15. Zhu, Y.; Cheng, Z.; Xiang, Q.; Chen, X.; Xu, J. Synthesis of functionalized mesoporous TiO_2-SiO_2 with organic fluoroalcohol as high performance DMMP gas sensor. *Sens. Actuators B: Chem.* **2017**, *248*, 785–792.
16. Cao, Y.; Fan, Y.; Ma, Z.; Cheng, Z.; Xiang, Q.; Duan, Z.; Xu, J. Urea-functionalized SBA-15 hybrids: Post-grafting synthesis, high-performance organophosphorus sensing and their response mechanism. *Sens. Actuators B: Chem.* **2018**, *273*, 1162–1169.
17. Xie, H.; Sun, X.X.; Yang, Q.; Wang, J.; Huang, Y. Novel gas sensor based on nano-zeolite films for the nerve agent simulant dimethylmethylphosphonate detection. In Proceedings of the 7th International Conference on Solid-State and Integrated Circuits Technology, Beijing, China, 18–21 October 2004, pp. 1719–1722.
18. Xie, H.; Hu, Q.; Zhou, J.; Huang, Y. The study of nanosized zeolite films in sensor for DMMP gas detection. In Proceedings of the 16th IEEE International Symposium on the Physical and Failure Analysis of Integrated Circuits; Suzhou, China, 6–10 July 2009; pp. 555–558.
19. Huang, J.; Jiang, Y.; Du, X.; Bi, J. A New Siloxane Functional Polymer for Chemical Vapor Sensor. In Proceedings of the 2010 Symposium on Photonics and Optoelectronics, Chengdu, China, 19–21 June 2010; pp. 1–5.
20. Qiao, Q.; Cui, X.; Xu, W.; Li, T.; Wu, Y. Fabrication and Properties of Self-Assembly ZrO_2 Film: In-Suit Detecting Atomized Chemical Warfare Agent Simulator Aqueous Solution With Higher Sensitivity. *IEEE Sens. J.* **2020**, *20*, 12166–12173.
21. Ha, S.; Lee, M.; Seo, H.O.; Song, S.G.; Kim, K.; Park, C.H.; Kim, I.H.; Kim, Y.D.; Song, C. Structural effect of thioureas on the detection of chemical warfare agent simulants. *ACS sensors* **2017**, *2*, 1146–1151.
22. Zheng, Q.; Zhu, Y.; Xu, J.; Cheng, Z.; Li, H.; Li, X. Fluoroalcohol and fluorinated-phenol derivatives functionalized mesoporous SBA-15 hybrids: High-performance gas sensing toward nerve agent. *J. Mater. Chem.* **2012**, *22*, 2263–2270.
23. Ramesh, S.; Lee, Y.-J.; Msolli, S.; Kim, J.-G.; Kim, H.S.; Kim, J.-H. Synthesis of a Co_3O_4@ gold/MWCNT/polypyrrole hybrid composite for DMMP detection in chemical sensors. *RSC Adv.* **2017**, *7*, 50912–50919.
24. Chung, Y.K.; Ha, S.; Woo, T.G.; Kim, Y.D.; Song, C.; Kim, S.K. Binding thiourea derivatives with dimethyl methylphosphonate for sensing nerve agents. *RSC Adv.* **2019**, *9*, 10693–10701.
25. Du, X.; Wang, Z.; Huang, J.; Tao, S.; Tang, X.; Jiang, Y. A new polysiloxane coating on QCM sensor for DMMP vapor detection. *J. Mater. Sci.* **2009**, *44*, 5872–5876.
26. Fan, G.; Wang, Y.; Hu, M.; Luo, Z.; Li, G. Synthesis of flowerlike nano-SnO_2 and a study of its gas sensing response. *Meas. Sci. Technol.* **2011**, *22*, 45203.
27. Guo, S.B.; Cheng, Z.X.; Zhu, H.Y.; Wang, L.Y.; Zhou, C.H. Hydrogen-bond acid group functionalized mesoporous-silica MCM-41 as sensing material to detect trace level organophosphorus vapor. In Proceedings of the Advanced Materials Research; T*rans. Tech. Publ.*, **2015**; *1092*, 780–783.
28. Kösemen, A.; Öztürk, S.; Şen, Z.; Kösemen, Z.A.; Harbeck, M.; Öztürk, Z.Z. Volatile organic compounds and dimethyl methyl phosphonate (DMMP) sensing properties of the metal oxide functionalized QCM transducers at room temperature. *J. Electrochem. Soc.* **2017**, *164*, B657.
29. Lee, Y.-J.; Kim, G.-H.; Jung, D.; Kim, J.-H. Dimethyl Methylphosphonate Detection Using a Quartz Crystal Microbalance as Chemical Warfare Sensor. *Nanosci. Nanotechnol. Lett.* **2015**, *7*, 1015–1018.
30. Lee, Y.-J.; Kim, J.-G.; Kim, J.-H.; Yun, J.; Jang, W.J. Detection of Dimethyl Methylphosphonate (DMMP) Using Polyhedral Oligomeric Silsesquioxane (POSS). *J. Nanosci. Nanotechnol.* **2018**, *18*, 6565–6569.
31. Li, W.; Wen, X.Y.; Li, S.M.; Wang, X.; Wang, J.Z.; Tang, H. Determination of DMMP using a polymer coated QCM sensor. In Proceedings of the Advanced Materials Research; Trans Tech Publ, 2012; Vol. 542, pp. 959–962.
32. Şen, Z.; Gürol, I.; Gümüş, G.; Musluoğlu, E.; Harbeck, M.; Ahsen, V.; Öztürk, Z.Z. Organophosphate sensing with vic-dioximes using QCM sensors. In Proceedings of the SENSORS, 2010 IEEE; IEEE, 2010; pp. 2127–2130.
33. Pei, Z.; Ma, X.; Ding, P.; Zhang, W.; Luo, Z.; Li, G. Study of a QCM dimethyl methylphosphonate sensor based on a ZnO-modified nanowire-structured manganese dioxide film. *Sensors* **2010**, *10*, 8275–8290.
34. Fan, G.; Wang, Y.; Hu, M.; Luo, Z.; Zhang, K.; Li, G. Template free synthesis of hollow ball-like nano-Fe_2O_3 and its application to the detection of dimethyl methylphosphonate at room temperature. *Sensors* **2012**, *12*, 4594–4604.
35. Hwang, H.M.; Hwang, E.; Kim, D.; Lee, H. Mesoporous non-stacked graphene-receptor sensor for detecting nerve agents. *Sci. Rep.* **2016**, *6*, 1–8.
36. Hwang, E.; Hwang, H.M.; Shin, Y.; Yoon, Y.; Lee, H.; Yang, J.; Bak, S.; Lee, H. Chemically modulated graphene quantum dot for tuning the photoluminescence as novel sensory probe. *Sci. Rep.* **2016**, *6*, 1–10.
37. Ying, Z.; Jiang, Y.; Du, X.; Xie, G.; Yu, J.; Wang, H. PVDF coated quartz crystal microbalance sensor for DMMP vapor detection. *Sens. Actuators B: Chem.* **2007**, *125*, 167–172.
38. Wang, Y.; Yang, Z.; Hou, Z.; Xu, D.; Wei, L.; Kong, E.S.W.; Zhang, Y. Flexible gas sensors with assembled carbon nanotube thin films for DMMP vapor detection. *Sens. Actuators B: Chem.* **2010**, *150*, 708–714.

39. Yoon, H.; Jang, J. Conducting-polymer nanomaterials for high-performance sensor applications: Issues and challenges. *Adv. Funct. Mater.* **2009**, *19*, 1567–1576.
40. Hatchett, D.W.; Josowicz, M. Composites of intrinsically conducting polymers as sensing nanomaterials. *Chem. Rev.* **2008**, *108*, 746–769.
41. Geng, L.; Wu, S. Preparation, characterization and gas sensitivity of polypyrrole/γ-Fe_2O_3 hybrid materials. *Mater. Res. Bull.* **2013**, *48*, 4339–4343.
42. Hamilton, S.; Hepher, M.J.; Sommerville, J. Polypyrrole materials for detection and discrimination of volatile organic compounds. *Sens. Actuators B: Chem.* **2005**, *107*, 424–432.
43. Joulazadeh, M.; Navarchian, A.H. Ammonia detection of one-dimensional nano-structured polypyrrole/metal oxide nanocomposites sensors. *Synth. Met.* **2015**, *210*, 404–411.
44. Kwon, O.S.; Park, C.S.; Park, S.J.; Noh, S.; Kim, S.; Kong, H.J.; Bae, J.; Lee, C.-S.; Yoon, H. Carboxylic acid-functionalized conducting-polymer nanotubes as highly sensitive nerve-agent chemiresistors. *Sci. Rep.* **2016**, *6*, 1–7.
45. Lama, S.; Kim, J.; Ramesh, S.; Lee, Y.-J.; Kim, J.; Kim, J.-H. Highly Sensitive Hybrid Nanostructures for Dimethyl Methyl Phosphonate Detection. *Micromachines* **2021**, *12*, 648.
46. Haghighi, E.; Zeinali, S. Nanoporous MIL-101 (Cr) as a sensing layer coated on a quartz crystal microbalance (QCM) nanosensor to detect volatile organic compounds (VOCs). *RSC Adv.* **2019**, *9*, 24460–24470.
47. Du, X.; Ying, Z.; Jiang, Y.; Liu, Z.; Yang, T.; Xie, G. Synthesis and evaluation of a new polysiloxane as SAW sensor coatings for DMMP detection. *Sens. Actuators B: Chem.* **2008**, *134*, 409–413.
48. Chevallier, E.; Scorsone, E.; Bergonzo, P. New sensitive coating based on modified diamond nanoparticles for chemical SAW sensors. *Sens. Actuators B: Chem.* **2011**, *154*, 238–244.
49. Joo, B.-S.; Huh, J.-S.; Lee, D.-D. Fabrication of polymer SAW sensor array to classify chemical warfare agents. *Sens. Actuators B: Chem.* **2007**, *121*, 47–53.
50. Wen, W.; Shitang, H.; Shunzhou, L.; Minghua, L.; Yong, P. Enhanced sensitivity of SAW gas sensor coated molecularly imprinted polymer incorporating high frequency stability oscillator. *Sens. Actuators B: Chem.* **2007**, *125*, 422–427.
51. Matatagui, D.; Martí, J.; Fernández, M.J.; Fontecha, J.L.; Gutiérrez, J.; Gràcia, I.; Cané, C.; Horrillo, M.C. Chemical warfare agents simulants detection with an optimized SAW sensor array. *Sens. Actuators B: Chem.* **2011**, *154*, 199–205.
52. Matatagui, D.; Fernandez, M.J.; Fontecha, J.; Santos, J.P.; Gràcia, I.; Cané, C.; Horrillo, M.C. Love-wave sensor array to detect, discriminate and classify chemical warfare agent simulants. *Sens. Actuators B: Chem.* **2012**, *175*, 173–178.
53. Raj, V.B.; Singh, H.; Nimal, A.T.; Sharma, M.U.; Gupta, V. Oxide thin films (ZnO, TeO_2, SnO_2, and TiO_2) based surface acoustic wave (SAW) E-nose for the detection of chemical warfare agents. *Sens. Actuators B: Chem.* **2013**, *178*, 636–647.
54. Singh, H.; Raj, V.B.; Kumar, J.; Mittal, U.; Mishra, M.; Nimal, A.T.; Sharma, M.U.; Gupta, V. Metal oxide SAW E-nose employing PCA and ANN for the identification of binary mixture of DMMP and methanol. *Sens. Actuators B: Chem.* **2014**, *200*, 147–156.
55. Raj, V.B.; Singh, H.; Nimal, A.T.; Tomar, M.; Sharma, M.U.; Gupta, V. Origin and role of elasticity in the enhanced DMMP detection by ZnO/SAW sensor. *Sens. Actuators B: Chem.* **2015**, *207*, 375–382.
56. Pan, Y.; Zhang, G.; Guo, T.; Liu, X.; Zhang, C.; Yang, J.; Cao, B.; Zhang, C.; Wang, W. Environmental characteristics of surface acoustic wave devices for sensing organophosphorus vapor. *Sens. Actuators B: Chem.* **2020**, *315*, 127986.
57. Grabka, M.; Jasek, K.; Pasternak, M. Application of polymethyl [4-(2, 3-difluoro-4-hydroxyphenoxy) butyl] siloxane in surface acoustic wave gas sensors for dimethyl methylphosphonate detection. *Sens. Actuators B: Chem.* **2021**, *329*, 129216.
58. Singh, H.; Raj, V.B.; Kumar, J.; Durani, F.; Mishra, M.; Nimal, A.T.; Sharma, M.U. SAW mono sensor for identification of harmful vapors using PCA and ANN. *Process. Saf. Environ. Prot.* **2016**, *102*, 577–588.
59. Islam, T.; Mittal, U.; Nimal, A.T.; Sharma, M.U. Surface Acoustic Wave (SAW) vapour sensor using 70 MHz SAW oscillator. In Proceedings of the 2012 Sixth International Conference on Sensing Technology (ICST); IEEE, 2012; pp. 112–114.
60. Chen, G.; Xie, X.; Wang, W.; He, S. An experimental research of love wave sensor based on $LiTaO_3/SiO_2$ structure. In Proceedings of the Proceedings of the 2014 Symposium on Piezoelectricity, Acoustic Waves, and Device Applications; Beijing, China, 30 October–2 November 2014; pp. 1–4.
61. Qi, X.; Liu, J.; Liang, Y.; Li, J.; He, S. The response mechanism of surface acoustic wave gas sensors in real time. *Jpn. J. Appl. Phys.* **2019**, *58*, 14001.
62. Wang, Y.; Du, X.; Long, Y.; Tang, X.; Tai, H.; Jiang, Y. The response comparison of a hydrogen-bond acidic polymer to sarin, soman and dimethyl methyl phosphonate based on a surface acoustic wave sensor. *Anal. Methods* **2014**, *6*, 1951–1955.
63. Cao, B.Q.; Huang, Q. Bin; Pan, Y.; Qin, M.L. The Respond Characteristic Property in Different temperatures of SAW Sensor with p-tertbutylcalix [4] arene Coatings to Detect DMMP. In Proceedings of the *Adv. Mater. Res.* **2014**, *1015*, 590–593.
64. Hartmann-Thompson, C.; Hu, J.; Kaganove, S.N.; Keinath, S.E.; Keeley, D.L.; Dvornic, P.R. Hydrogen-bond acidic hyperbranched polymers for surface acoustic wave (SAW) sensors. *Chem. Mater.* **2004**, *16*, 5357–5364.
65. Pan, Y.; Guo, T.; Zhang, G.; Yang, J.; Yang, L.; Cao, B. Detection of organophosphorus compounds using a surface acoustic wave array sensor based on supramolecular self-assembling imprinted films. *Anal. Methods* **2020**, *12*, 2206–2214.
66. Hartmann-Thompson, C.; Keeley, D.L.; Voit, B.; Eichhorn, K.; Mikhaylova, Y. Hyperbranched polyesters with internal and exo-presented hydrogen-bond acidic sensor groups for surface acoustic wave sensors. *J. Appl. Polym. Sci.* **2008**, *107*, 1401–1406.

67. Kim, E.; Kim, J.; Ha, S.; Song, C.; Kim, J.-H. Improved Performance of Surface Acoustic Wave Sensors by Plasma Treatments for Chemical Warfare Agents Monitoring. *J. Nanosci. Nanotechnol.* **2020**, *20*, 7145–7150.
68. Xu, S.; Zhang, R.; Cui, J.; Liu, T.; Sui, X.; Han, M.; Zheng, F.; Hu, X. Surface Acoustic Wave DMMP Gas Sensor with a Porous Graphene/PVDF Molecularly Imprinted Sensing Membrane. *Micromachines* **2021**, *12*, 552.
69. Wang, W.; He, S.; Li, S.; Liu, M.; Pan, Y. Advances in SXFA-coated SAW chemical sensors for organophosphorous compound detection. *Sensors* **2011**, *11*, 1526–1541.
70. Long, Y.; Wang, Y.; Du, X.; Cheng, L.; Wu, P.; Jiang, Y. The different sensitive behaviors of a hydrogen-bond acidic polymer-coated SAW sensor for chemical warfare agents and their simulants. *Sensors* **2015**, *15*, 18302–18314.
71. Dinca, V.; Viespe, C.; Brajnicov, S.; Constantinoiu, I.; Moldovan, A.; Bonciu, A.; Toader, C.N.; Ginghina, R.E.; Grigoriu, N.; Dinescu, M. MAPLE assembled acetylcholinesterase–polyethylenimine hybrid and multilayered interfaces for toxic gases detection. *Sensors* **2018**, *18*, 4265.
72. Kim, J.; Park, H.; Kim, J.; Seo, B.-I.; Kim, J.-H. SAW Chemical Array Device Coated with Polymeric Sensing Materials for the Detection of Nerve Agents. *Sensors* **2020**, *20*, 7028.
73. Wang, Y.; Du, X.; Li, Y.; Long, Y.; Qiu, D.; Tai, H.; Tang, X.; Jiang, Y. A simple route to functionalize siloxane polymers for DMMP sensing. *J. Appl. Polym. Sci.* **2013**, *130*, 4516–4520.
74. Nieuwenhuizen, M.S.; Harteveld, J.L.N. A surface acoustic wave gas sensor for organophosphorus compounds. *Sens. Actuators B: Chem.* **1994**, *19*, 502–505.
75. Sayago, I.; Matatagui, D.; Fernández, M.J.; Fontecha, J.L.; Jurewicz, I.; Garriga, R.; Muñoz, E. Graphene oxide as sensitive layer in Love-wave surface acoustic wave sensors for the detection of chemical warfare agent simulants. *Talanta* **2016**, *148*, 393–400.
76. Segal, S.R.; Suib, S.L.; Tang, X.; Satyapal, S. Photoassisted decomposition of dimethyl methylphosphonate over amorphous manganese oxide catalysts. *Chem. Mater.* **1999**, *11*, 1687–1695.
77. Collins, G.E.; Buckley, L.J. Conductive polymer-coated fabrics for chemical sensing. *Synth. Met.* **1996**, *78*, 93–101.
